# Enhancing the Bioavailability and Bioactivity of Curcumin for Disease Prevention and Treatment

**DOI:** 10.3390/antiox13030331

**Published:** 2024-03-08

**Authors:** Caroline Bertoncini-Silva, Adelina Vlad, Roberta Ricciarelli, Priscila Giacomo Fassini, Vivian Marques Miguel Suen, Jean-Marc Zingg

**Affiliations:** 1 Department of Internal Medicine, Division of Nutrology, Ribeirão Preto Medical School, University of São Paulo, Ribeirão Preto 14049-900, SP, Brazil; bertoncinicaroline@usp.br (C.B.-S.); priscilafassini@usp.br (P.G.F.); 2 Department of Functional Sciences I/Physiology, Faculty of Medicine, Carol Davila University of Medicine and Pharmacy, 050474 Bucharest, Romania; adelina.vlad@umfcd.ro; 3 Department of Experimental Medicine, University of Genoa, 16132 Genoa, Italy; ricciarelli@medicina.unige.it; 4 IRCCS Ospedale Policlinico San Martino, 16132 Genoa, Italy; 5 Department of Biochemistry and Molecular Biology, Miller School of Medicine, University of Miami, Miami, FL 33136, USA

**Keywords:** Curcumin, curcuminoids, nanoformulation, bioavailability, bioactivity, neurodegeneration, cardiovascular disease, obesity, prevention, therapy

## Abstract

Curcumin, a natural polyphenolic component from *Curcuma longa* roots, is the main bioactive component of turmeric spice and has gained increasing interest due to its proposed anti-cancer, anti-obesity, anti-inflammatory, antioxidant, and lipid-lowering effects, in addition to its thermogenic capacity. While intake from dietary sources such as curry may be sufficient to affect the intestinal microbiome and thus may act indirectly, intact curcumin in the body may be too low (<1 microM) and not sufficient to affect signaling and gene expression, as observed in vitro with cultured cells (10–20 microM). Several strategies can be envisioned to increase curcumin levels in the body, such as decreasing its metabolism or increasing absorption through the formation of nanoparticles. However, since high curcumin levels could also lead to undesired regulatory effects on cellular signaling and gene expression, such studies may need to be carefully monitored. Here, we review the bioavailability of curcumin and to what extent increasing curcumin levels using nanoformulations may increase the bioavailability and bioactivity of curcumin and its metabolites. This enhancement could potentially amplify the disease-preventing effects of curcumin, often by leveraging its robust antioxidant properties.

## 1. Introduction

The rhizome of turmeric (*Curcuma longa*) has been traditionally used in Asian countries as color, spice (e.g., in curry) and for the prevention of a number of diseases. As the main bioactive component of turmeric, curcumin is a polyphenol that is relatively unstable in aqueous solution, has low bioavailability to the organs of the human body, and, upon uptake, is rapidly converted to a number of bioactive metabolites. In traditional topical applications, curcumin has shown benefits for skin diseases, infections, and wound healing, whereas more recent scientific studies with cell culture and animals indicate preventive and even therapeutic activity in diseases such as cancer, inflammation, obesity, atherosclerosis, diabetes, dyslipidemia, liver diseases such as nonalcoholic steatohepatitis, inflammatory bowel disease (IBD), various disorders of the eye, and age-related neurodegeneration (Alzheimer’s and Parkinson’s disease) [1,2,3,4,5].

**Figure 1:**
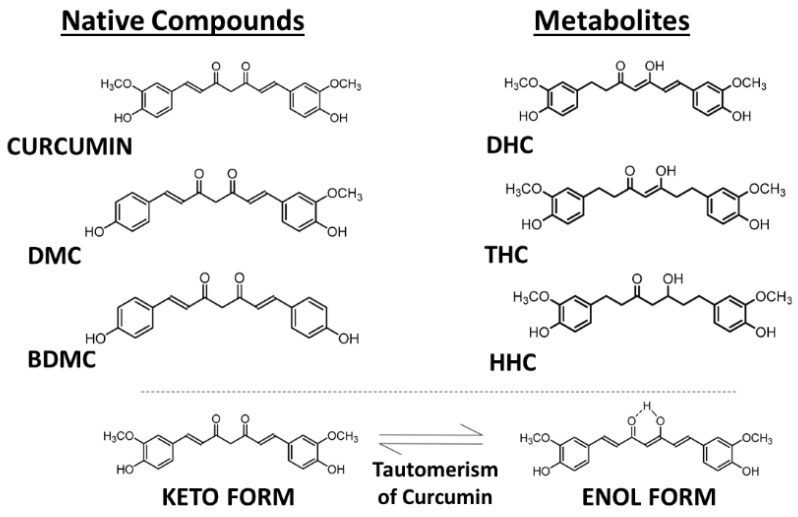
Molecular structures of curcuminoids—curcumin, demethoxycurcumin (DMC), and bisdemethoxycurcumin (BDMC) and the principal metabolites—dihydrocurcumin (DHC), tetrahydrocurcumin (THC), and hexahydrocurcumin (HHC). Tautomerism of curcuminoids. Source: authors’ original work.

Curcumin, also known as diferuloylmethane (International Union of Pure and Applied Chemistry (IUPAC) name (1*E*,6*E*)-1,7-bis(4-hydroxy-3-methoxyphenyl)-1,6-heptadiene-3,5-dione), is present in dried turmeric powder from the rhizome of *Curcuma longa* L. at a concentration of 2–5%. In commercially available turmeric powder, the major bioactive components are curcumin and another two chemically related compounds, demethoxycurcumin (DMC) and bisdemethoxycurcumin (BDMC), which are present in different relative amounts, with curcumin making up about 77%, DMC 17%, and BDMC 3% (Figure 1) [6]. These curcuminoids show similar cellular regulatory effects but often different potencies, most likely as a result of different uptake and metabolism in the body. For example, the three analogs show different relative anti-oxidative, anti-inflammatory, and anti-angiogenic activities [7]. In addition to functioning as a chemical antioxidant, curcumin can act as a Michael acceptor and as a metal chelator—as a hydrophobic small molecule it can interact in cells with many lipophilic binding sites in proteins and lipid domains and modulate their activity in a more or less specific manner [8].

Curcuminoids are regarded as safe, as outlined by the Food and Drug Administration Office of Food Additive Safety (Agency Response Letter GRAS Notice No. Grn 000822. U.S. Food and Drug Administration, 2019) [9]. In cultured cells, curcumin affects several cellular functions and some of the molecular targets have been identified; however, these events often occur only at concentrations that cannot be easily achieved in vivo. As discussed in this review, the low bioavailability of curcumin has prompted the development of formulations that lead to higher levels of curcumin and/or its metabolites in the body, using strategies that reduce its metabolism or increase its uptake and stability by incorporating curcumin into nanoformulations. It is often still unknown whether the consequent higher level of curcuminoids in the body is associated with enhanced regulatory effects. Animal models and human clinical trials are used to test these nanoformulations for increased bioavailability, bioactivity, and disease-preventing activity. For human usage, these formulations need also to be tested for potential adverse or cytotoxic side effects [1,2,3,4,5].

During absorption through intestinal epithelial cells, most of the free curcumin becomes conjugated, forming the more water-soluble curcumin glucuronide and sulfate [10,11]. In addition to DMC and BDMC, a number of further metabolites of curcumin are formed upon uptake into cells, but they are often unstable and present only transiently, such as dihydrocurcumin (DHC), dihydrobisdemethoxycurcumin (DHBC), tetrahydrocurcumin (THC), tetrahydrobisdemethoxycurcumin (THBC), tetrahydrodemethoxycurcumin (THDC), hexahydrocurcumin (HHC), hexahydrobisdemethoxycurcumin (HHBC), hexahydrodemethoxycurcumin (HHDC), and octahydrocurcumin (OHC) (Figure 1) [7]. Unmetabolized curcumin in the blood is present at extremely low concentrations, but low amounts of THC can be detected in the plasma after intraperitoneal injection of mice with curcumin [10,11,12].

**Figure 2:**
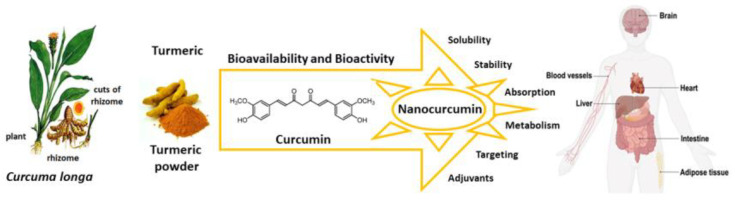
Curcumin nanoformulation strategies enhance bioavailability and bioactivity, and improve solubility, stability, absorption, and targeting of specific cells. Source: authors’ original work.

Here we review the bioavailability of curcumin and to what degree nanoformulations may enhance the bioactivity of curcumin and its metabolites as preventive and curative agents against several diseases that are susceptible to its antioxidant actions (Figure 2).

## 2. Curcumin and Curcuminoids

### 2.1. Comparative Analysis of Curcumin Levels in the Body versus Cell Culture

To ensure the effective exertion of its biological activity, curcumin’s absorption and distribution in body tissues should be considered. According to Ryu et al. [13], the majority of curcumin is metabolized in the liver and intestine, with only a small quantity remaining detectable in other organs. Depending on the solvent polarity, pH, and temperature, curcumin tautomerization can occur, with the keto form being more stable and having antioxidant properties, whereas the enol form can act as a prooxidant and undergo more rapid degradation [8,14].

In most investigations, curcumin is administered orally, whether in humans or animals. However, evidence suggests that when administered intraperitoneally—entailing the injection of the substance into the peritoneum, a body cavity—curcumin exhibits higher bioavailability compared to gavage (oral administration). It is worth noting that the intraperitoneal route is more commonly used in animal studies than in human research [15].

#### 2.1.1. Curcumin Levels in Serum and Plasma

Marczylo et al. reported that, two hours following oral administration of curcumin to rats at a dose of 340 mg/kg body weight, the compound was present in plasma at a concentration of 16.1 ng/mL, in the intestinal mucosa at a concentration of 1.4 mg/g, and in the liver at a concentration of 3.7 ng/g [16].

Notably, in a separate investigation, oral administration of curcumin to rats at a dose of 2 g/kg body weight resulted in a peak serum concentration of 1.35 ± 0.23 µg/mL within 0.83 h, whereas, when the same dose was given to humans, it led to undetectable or extremely low serum levels or curcumin (0.006 ± 0.005 µg/mL) after one hour [17]. 

In mice, a dose of 0.1 g/kg body weight administered intraperitoneally resulted in a maximum plasma concentration of free curcumin of 2.25 µg/mL within the first 15 min. When administered orally, in one hour, the maximum concentration reached was 0.22 μg/mL, and subsequently decreased to below 5 ng/mL within 6 h [12]. However, in humans, serum levels of curcumin remained undetectable even after the administration of high doses (up to 8 g/day, orally). When given in higher doses (10 and 12 g/day), low levels of curcumin were detected: 50.5 ng/mL and 51.2 ng/mL, respectively [18,19]. 

Sharma et al. conducted a study in which rats were administered a high dose of curcumin (1.2 g/kg body weight) for 14 days, and they detected low nanomolar levels of curcumin in the plasma (ranging from 0 to 12 nM) [20]. When curcumin was administered at a dose of 2 g/kg body weight in rats, peak serum concentrations of 1.00 ± 0.26 pg/mL were rapidly attained within 0.75 h and remained plateaued for up to one hour [17].

In a Phase I clinical trial assessing curcumin, oral administration of daily doses at 4, 6, and 8 g yielded average serum concentrations of 0.51 ± 0.11 µM, 0.63 ± 0.06 µM, and 1.77 ± 1.87 µM, respectively, within a 12 h timeframe [21]. In another clinical trial, the ingestion of 3.6 g of curcumin daily resulted in a plasma curcumin level of 11.1 nmol/L after one hour of dosing [22]. 

Discrepancies identified in the results can be attributed to variations, such as differences in experimental conditions, including distinct treatment periods, diverse doses, and different modes of curcumin administration, among other factors.

#### 2.1.2. Curcumin Levels in the Intestine

A recent study reports high concentrations of curcumin detected in the gastrointestinal tract of rats following oral administration [23]. Despite being almost completely insoluble in water, curcumin demonstrates stability in the acidic pH of the stomach, leading to slower degradation, with less than 20% of the total curcumin decomposed at one hour [24,25]. Additionally, a high dose of curcumin (1.2 g/kg body weight) administered to rats for 14 days revealed concentrations ranging from 0.2 to 1.8 µmol/g in colonic mucosal tissue [20].

In mice, when administered intraperitoneally at a dose of 100 mg/kg, curcumin reached peak levels of 200 nmol/g in the small intestinal mucosa within 2 h [26]. Furthermore, an orally administered dose of 0.1 g/kg body weight resulted in a curcumin concentration in the mice intestine of 177.04 µg/g one hour after administration [12].

Garcea et al. orally administered 3.600 mg/day of curcumin to humans and reported a colorectal concentration of 12.7 nmol/g within 38 min [27]. In another investigation involving individuals undergoing colorectal endoscopy, the levels of curcuminoids were quantified in the colorectal mucosa. Oral administration of curcumin at a dose of 2.35 g/day resulted in tissue biopsies showing 48.4 mg/g (127.8 nmol/g) of curcumin, which persisted in the mucosa for up to 40 h after administration [28]. 

#### 2.1.3. Curcumin Levels in Cultured Cells

When analyzed in a cell culture medium, research shows that curcumin is more stable in the presence of 10% fetal calf serum or in human blood, exhibiting less than 20% decomposition in one hour. In contrast, in serum-free medium, curcumin undergoes 90% decomposition in 30 min [25].

A study carried out by Shoji et al. examined the levels of curcumin in HepG2 cells. After treating the cells with 25 µM curcumin for 24 h, high concentrations of curcumin were observed (1840–5650 pmol of curcumin/2.5 × 10^6^ cells) in HepG2 cells after 1–9 h of incubation [11].

Muangnoi et al. (2019) investigated levels (bioavailable fraction) derived from 5 µM of curcumin permeating Caco-2 cells (2.5 × 10^4^ cells) at different time intervals. The authors observed that the amount of curcumin in the bioavailable fraction at 15 min was 0.013 µM, with a gradual increase over time, reaching maximum concentrations of 0.055 µM at 60 min. The remaining amount at 4 h was only 0.031 µM [29]. In human THP-1 monocytes and macrophages, curcumin is readily taken up and slowly metabolized mainly to hexahydrocurcumin sulfate (HHCS), and the metabolites are released into the cell culture medium [30]. In contrast, THC is slowly absorbed and rapidly converted to secondary metabolites (e.g., HHCS). Differential uptake and metabolism of curcumin and THC may thus explain their distinct regulatory effects on lipid uptake that was observed in THP-1 macrophages.

To summarize the information, Table 1 below includes the data found regarding the bioavailability of curcumin in animal models and humans.

According to the information in the table above, it is evident that curcumin, when administered orally, has much lower bioavailability, compared to the intraperitoneal and intravenous routes, showing the important role played by the route of administration. Further, as reviewed in the following, several strategies have been investigated and developed that increase the levels of curcumin in the body.

### 2.2. Increasing the Bioavailability of Curcumin with Nanoformulations

Similar to other polyphenols, the bioavailability of curcumin from the diet and even from intravenous administration routes is low, and sub-micromolar concentrations can be reached only transiently. During uptake, curcumin is inefficiently transported across the intestinal epithelium, rapidly metabolized, and eliminated. Further, it is inherently unstable in aqueous solution, in particular at a neutral/basic pH, where the enol state of curcumin is formed [25,31]. Therefore, a myriad of nanoformulations have been developed that either lead to a systemic increase in curcumin or are targeted to specific cells, tissues, or organelles (Table 2). Nanoparticles containing curcumin have also been generated from extracts of raw turmeric rhizome as starting material without added nanocarriers [32]. The design principles and methods by which these curcumin nanoformulations are formed have been extensively reviewed and will not be addressed here [33,34].

The pharmaceutical strategies by which curcumin becomes more bioavailable and bioactive involve nanoformulations with increased solubility, improved stability during gastrointestinal uptake, altered absorption route, and coadministration with adjuvants that inhibit the detoxification enzymes, such as piperine (Table 3) [33]. Nanoformulations can increase the levels of curcumin in various tissues, including various parts of the brain, suggesting transport across the blood–brain barrier (BBB) [34,44,48].

The stability of curcumin can be affected by nanoformulation, e.g., when encapsulated into vesicles of soy lecithin of about 100 nm in diameter, wherein a synergistic effect of concentration and confinement was observed as a result of keto to enol tautomerization [14]. Furthermore, once in circulation, curcumin may become stabilized by binding to serum albumin that may, similarly to vitamin E, facilitate its transport across the vasculature into tissues and cells [7,49,50]. Curcumin is more stable in a cell culture medium containing 10% fetal calf serum as well as in human blood; 50% of curcumin is still intact after 8 h of incubation [25]. Interestingly, the ability of binding to albumin and consequently being uptaken into cells were different for curcumin, BDMC, and DMC, which may explain, in part, their different biological activities [7,51]. Accordingly, a number of formulations of curcuminoids enhanced the bioavailability of total curcuminoids in humans (7- to 30-fold), leading to measurable curcumin concentration in plasma (~22 ng/mL), and some of these formulations increased BDMC and DMC bioavailability even better than that of curcumin (up to 68-fold) [31].

The latest generation of curcumin nanoformulations can increase the bioavailability of free curcumin in plasma greater than 100-fold and have superior absorption, cellular uptake, BBB permeability, and tissue distribution [33]. A review of 11 curcumin nanoformulations tested in clinical studies showed increased relative bioavailability (RB) ranging from 9- to 185-fold, but an analysis of the design of these studies, curcumin dosage, administration, analytical method, pharmacokinetic parameters, and the composition of study population revealed differences that make it difficult to compare these RB numbers directly [52]. Not only the composition of nanoparticles affects the bioavailability, but also particle size and route of administration [53], e.g., for curcumin prepared as solid dispersions, a smaller particle size increased the bioavailability when administered orally, whereas a larger particle size increased the bioavailability when administered intravenously [54]. 

Nanoformulations may prevent diseases with an inflammatory, oxidative stress-, or aging-related component including cardiovascular, liver, lung, neurodegenerative, cancer, metabolic, and gastrointestinal diseases (Table 1) [33,34,37,38,39,46,48,55,56]. 

These diseases often have an aging-related component and may be targeted with nanoformulations of curcumin that do not only act as senolytics but also induce senescence in malignant and normal cells [57]. Mechanistically, curcumin may reduce reactive oxygen species (ROSs) and reduce lipid peroxidation by acting as an antioxidant and/or by modulating signal transduction and the expression of genes that are relevant for inflammation and lipid homeostasis [58,59]. Liposomal curcumin facilitates the polarization of pro-inflammatory M1 to anti-inflammatory M2 macrophages, which is relevant for the treatment of a variety of inflammatory conditions [60,61]. Further, curcumin can modulate cellular lipid homeostasis, leading to the inhibition of lipid accumulation and reduced inflammatory gene expression, steatohepatitis, and macrophage foam cell formation, which are relevant for cardiovascular disease [10,58,59,62,63,64]. When formulated as nanoparticles, such as polymeric curcumin–bioperine–PLGA (poly (D,L-lactic-co-glycolic acid)ester terminated), these activities can be enhanced [42]. High-fat diet-induced adiposity was alleviated by dietary curcumin (0.2%) in C57BL/6 mice by improving insulin sensitivity, inflammatory response in white adipose tissue (WAT), enhanced metabolic activity of brown adipose tissue (BAT), and whole-body energy metabolism via the FNDC5/p38 MAPK/ERK pathways. The isomerization of curcumin to cis-trans curcumin increased its binding to the adenosine receptors A_2A_ and A_2B_, and incorporating cis-trans curcumin into nanoformulations may be a strategy to facilitate potential therapeutic inhibitory effects on inflammation and inflammatory pain [65].

Another strategy to enhance the bioactivity of curcumin is to present it in combination with other dietary bioactive compounds that were reported to lead to sufficient amounts in the body to affect gene expression even without nanoformulation. In transgenic mice carrying an electrophile response element (EpRE)–luciferase reporter construct, dietary curcumin, in combination with other dietary bioactive compounds, was able to activate nuclear factor erythroid 2-related factor 2 (Nrf2), showing that, despite its low bioavailability, dietary curcumin can have cellular regulatory effects in vivo. Furthermore, intraperitoneal injections of curcumin induced EpRE-dependent promoter activity in the intestine, liver, kidney, and spleen. When administered orally, a combination of turmeric extracts (containing curcumin) with extracts made of coffee, thyme, broccoli, rosemary, and red onion fed orally, induced EpRE-mediated luciferase in lung and adipose tissue, suggesting that sufficient curcumin levels can be achieved for organ-specific induction of antioxidant defense by curcumin [66].

For nanoformulations with increased bioavailability, adverse side effects that can come from the higher level of curcumin itself, or the other components in the formulation can be of concern: however, as recently reviewed, most of the curcumin formulations studied did not show issues with safety, tolerability, or efficacy in clinical trials [33].

**Table 3:** Curcumin formulations with increased bioavailability in humans [52].

Formulation	Composition	Bioavailability Increase	Reference
Micronized curcumin with turmeric essential oils (BCM-95CG) (Biocurcumax)	Combination of curcuminoids with volatile oils of turmeric rhizome, which are usually eliminated during preparation.	7-fold, compared to 6.4-fold with curcumin−lecithin−piperine.	[67]
Curcumin as solid lipid particles (LONGVIDA, M3C-X)	Solid lipid curcumin particles (SLCPs) with soy lecithin containing purified phospholipids, docosahexaenoic acid (DHA), and/or vegetable stearic acid, ascorbyl (vitamin C) esters, and inert ingredients.	Plasma concentrations of 22.43 ng/mL after 2.4 h, compared to undetectable levels without formulation.	[68]
Colloidal nanoparticles dispersed with a high-pressure homogenizer (THERACURMIN)	Nanoparticle colloidal dispersion, prepared with gum ghatti and glycerine, consisting of 10 *w*/*w*% of curcumin, 2% of other curcuminoids such as demethoxycurcumin and bisdemethoxycurcumin, 46% of glycerin, 4% of gum ghatti, and 38% of water.	Tmax and AUC_0–6 h_ values for total curcumin: 27.6-fold increased stability, water-solubility, stable preparation, and enhanced gastrointestinal absorption.	[69]
A 95% soybean-based phospholipid−curcumin formulation (Meriva)	Lipophilic matrix composed of curcumin/soybean lecithin/microcrystalline cellulose (1:2:2), 43 mg per capsule: curcumin (33 mg), demethoxycurcumin (8 mg), and bisdemethoxycurcumin (1 mg)— total curcuminoids 42 mg.	Increased absorption of curcumin and total curcuminoids by 19- and 32-fold, respectively. Increased absorption of DMC by 68-fold and of BDMC by 57-fold; hydrolytic stabilization of curcumin at intestinal pH may increase the curcumin load for the gut microbiota.	[70]
Encapsulated curcumin	Inner coating material of microcapsules was constituted by cellulose derivative (Ethocel 100) as a first layer and hydrogenated vegetable oil (HVO) as an external layer.	Curcuminoid microencapsulation increased bioavailability from enriched bread, probably preventing biotransformation.	[71]
Micronized curcumin powder	Micronized powder and particularly liquid micellar formulation of curcumin.	Oral bioavailability with AUC values in plasma for total curcumin 9- and 185-fold, respectively. Higher concentrations of DMC and BDMC were also observed.	[72]
Comparison of formulations of curcumin: CHC (cellulosic derivatives with antioxidants) CC (cellulosic derivatives with γ- cyclodextrin) CP (phytosome formulation) CTR (formulation with volatile oils of turmeric rhizome)	Combinations of a hydrophilic carrier.	The total concentration of curcuminoids in the new formulations was higher (45.9-fold in CHC and 37.4-fold in CC) than in CP (7.9- to 8.4-fold) and CTR (1.2- to 1.3-fold).	[73,74]
Curcumin solid dispersion (C-SD)	Large particle sizes of C-SDs were pulverized using zirconia beads.	Particle size determined the uptake, with an increased uptake of small particles orally, and an increased uptake of large particles intravenously in rats.	[54]

### 2.3. Antimicrobial Action of Curcumin and Curcumin Nanoformulations

Curcumin is well known as an antimicrobial molecule against a wide spectrum of both gram-positive and -negative bacteria, but with a minimal inhibitory concentration (MIC) of about 100–500 µg/mL, antimicrobial applications within the body may be difficult to achieve [35,75]. However, the antimicrobial action of curcumin may be useful for topical applications such as against skin infections, or for oral and intestinal treatments where higher levels can be achieved and are of less concern for negative side effects (Table 2). In topical applications, the curcumin metabolite tetrahydrocurcumin (THC) may be a better option, as it does not lead to any coloration [76,77]. Further, curcumin can prevent the growth of bacteria on foods and thus prevent infection indirectly [78]. Combination of curcumin with (-)-epigallocatechin gallate (EGCG), the most active component of tea, was more efficient in inhibiting biofilm formation by a number of wastewater bacteria [79]. In addition to direct bactericidal/static effects, mechanisms of action include cell membrane disruption, interference with quorum sensing, inhibition of biofilm formation, inhibition of cell division, induction of oxidative stress, induction of programmed cell death, phototoxicity, modulation of bacterial cell metabolism, and inhibition of the intracellular proliferation of bacteria [35,75,80]. The antimicrobial action of curcumin against *Salmonella* also involves reducing the number of flagella by directly binding to them and making them unstable [81]. However, as some studies suggested, curcumin can also interfere with the antimicrobial action of certain antibiotics and of γ-interferon, most likely as a result of scavenging reactive oxygen species (ROS) at all places that are produced in the infected cells as part of their bactericidal mechanism of action [82,83].

Recent research has shown that nanoformulations of curcumin can have increased antimicrobial activity in these applications, since relatively high levels of curcumin can be achieved [35,40,75,84]. Alternative or additional ways to enhance the anti-microbial and biofilm inhibitory activity are based on co-treatment with a number of other agents, including antibiotics [85], honey [86], or other polyphenols [66]. Further, it appears possible that anti-microbial curcumin nanoformulations could be designed that target selectively pathogenic bacteria in the skin or intestine (instead of current formulations that target eukaryotic cells, e.g., tumors or macrophages) [87,88].

The interplay between microbiota and curcumin and the generated metabolites is thought to promote a healthy condition in the human body that goes far beyond the direct effects at the intestinal epithelium [89,90]. Curcumin can positively alter microbial diversity and enrich mostly beneficial bacterial strains, leading to improved intestinal barrier function and less inflammation [90,91]. The degree of biotransformation by the intestinal microbiome may influence the cellular effects in the body and a unique enzyme in *E. coli*, the NADPH-dependent curcumin/dehydrocurcumin reductase (CurA) was reported to metabolize curcumin to DHC and THC [92,93]. Accordingly, in a rat model of high-fat diet-induced non-alcoholic steatohepatitis (NASH), improvement in hepatic lipid accumulation, inflammation, and endothelial dysfunction correlated with the formation of THC and was weakened after inhibiting bacterial growth and THC formation using antibiotic treatment [94]. Whether increased levels of THC can be generated in the intestine with curcumin nanoformulations remains to be demonstrated.

### 2.4. Regulatory Effects of Curcumin and Curcumin Nanoformulations in Different Tissues

#### 2.4.1. Regulatory Effects of Curcumin in the Intestine and on the Microbiome

Due to the low systemic bioavailability of curcumin, its clinical application is limited, and it is difficult to understand its pharmacological effects in vivo [95]. However, a hypothesis has recently emerged that curcumin could exhibit direct regulatory effects, mainly in the gastrointestinal tract/intestinal mucosa, since high concentrations of this substance were detected after oral or intraperitoneal administration (Table 2) [96,97].

**Figure 3:**
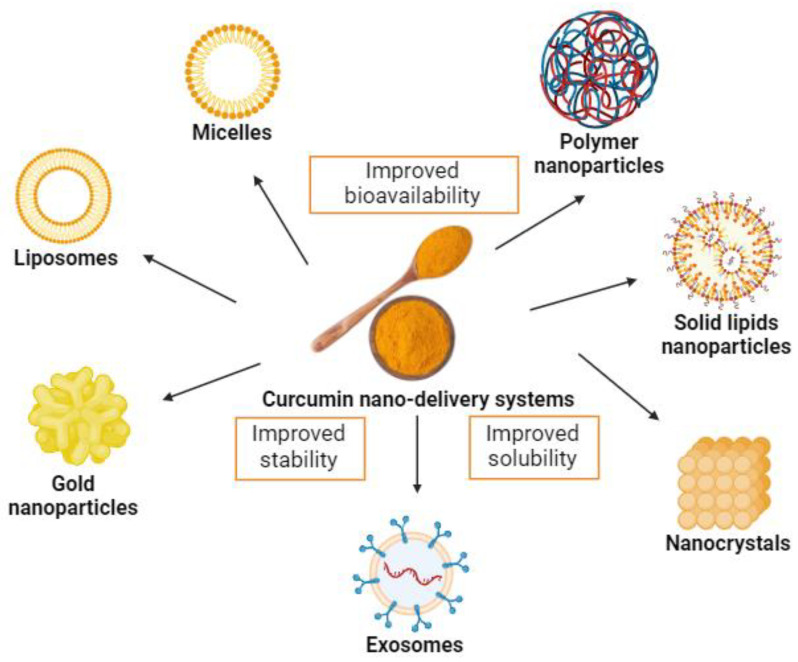
Examples of curcumin nano-delivery systems. Source: authors’ original work.

Although part of the metabolism of curcumin occurs in the intestine, strategies aimed at increasing its bioavailability and biological activity are substantially relevant. Hence, new and interesting strategies have emerged to increase oral curcumin bioavailability, including nanotechnology-based systems, such as micelles, liposomes, exosomes, phospholipid complexes, nanoemulsions, nanostructured lipid carriers, and biopolymer nanoparticles [56,98,99] (Figure 3). Moreover, these systems present potential benefits of possible cellular targeting and improvement in cellular uptake [100].

Ohno et al. investigated the effects of nanoparticle curcumin (called Theracurmin) on experimental colitis in mice and found that treatment with Theracurmin suppressed the development of dextran sulfate sodium (DSS)-induced colitis, especially through modulation of the gut microbiota. Data show that the application of nanotechnology for curcumin has notably improved its water solubility and oral bioavailability, facilitating absorption from the gut [101]. 

Recently, a study carried out on mice demonstrated that the supplementation of nanobubble curcumin extract had a beneficial effect on health and improved gut microbiota composition [102]. 

Sun et al. demonstrated that mice treated with curcumin complexed with exosomes were protected against lipopolysaccharide (LPS)-induced septic shock. These data show that an increase in the stability of curcumin in vitro and bioavailability in vivo also happens because of the formation of exosome–curcumin complexes [103].

A study conducted by Wang et al. (2021) showed that in an animal model, curcumin liposomes (Curcumin-LPs) alleviated clinical symptoms such as weight loss, diarrhea, and fecal bleeding in DSS-induced ulcerative colitis. In addition, it could also prevent colon tissue damage and colon shortening, as well as reduce the production of malondialdehyde (MDA), colonic myeloperoxidase (MPO), interleukin-6 (IL-6) and tumor necrosis factor-α (TNF-α) [104].

Another study compared three types of nanocarriers based on curcumin-loaded lipids in the treatment of IBD in a murine DSS-induced colitis model. The authors found that, in vitro, two self-nanoemulsifying drug delivery systems (SNEDDS) and nanostructured lipid carriers (NLC) of the three models used, significantly reduced TNF-α secretion by LPS-activated macrophages. In vivo, only nanostructured lipid carriers (NLC) were effective in reducing neutrophil infiltration and TNF-α secretion and, thus, colonic inflammation [105]. The authors hypothesized that patients with IBD lack some physiologically important lipids. And, because some lipids have immunomodulatory properties, it is hypothesized that the combination of lipids and anti-inflammatory drugs in a nanocarrier may be an important strategy in the treatment of IBD [105].

In an in vitro study, the authors evaluated the activity of curcumin against cell lines of colorectal cancer, and they observed that the incorporation of curcumin in liposomes improved absorption in the gastrointestinal tract, thus enhancing its antitumor activity, mainly because this formulation seems to be potent against intestinal epithelial cancer cell lines, HCT116 and HCT15 (cell lines expressing the multidrug-resistant (MDR) phenotype) [106].

In addition to the previously mentioned strategies, several natural agents have also been used to increase the bioavailability of curcumin [107]. Among these, the most used is piperine (the main active component of black pepper) [17,108,109,110], which increases the bioavailability of curcumin by blocking biotransformation [111].

In a clinical study, the authors found that 2 g of curcumin administered concomitantly with 20 mg of piperine, an inhibitor of hepatic and intestinal glucuronidation, appeared to promote a significant 2000% increase in the oral bioavailability of curcumin. In rats, when administered alone at a dose of 2 g/kg per rat, curcumin achieved moderate serum concentrations within 4 h. When administered concomitantly, piperine at a dose of 20 mg/kg increased the serum concentration of curcumin in a shorter time (1–2 h after administration). While curcumin is one of the most extensively studied plant secondary metabolites, the majority of the studies were performed in vitro or on animals [112]. When analyzed in humans, 2 g of isolated curcumin showed undetectable or very low serum levels. After the concomitant administration of 20 mg of piperine, an increase in concentrations was observed within the time frame of 0.25 to 1 h after administration [17].

Zeng et al. analyzed whether pre-administration of piperine interfered with the oral bioavailability of curcumin. Initially, the rats received 20 mg/kg of piperine followed by 200 mg/kg of curcumin at different time intervals (between 0.5 and 8 h) after piperine administration. The authors observed that, in those rats in which piperine pre-administration was performed before receiving curcumin, there was a significant increase in the oral bioavailability of curcumin, especially at 6 h after piperine administration [113].

Shi et al. evaluated the effects of the combined use of curcumin and piperine on performance, intestinal barrier function, and antioxidant capacity of weaned piglets. The data demonstrate that the jejunum and ileum villus height, the villus height/crypt depth ratio, and the mRNA expression levels of occludin, claudin-1, and zonula occluden-1 in jejunal and ileal mucosa were higher in the curcumin and piperine group, thereby improving the permeability [114].

To achieve the combinational anti-cancer effect in the in vitro model of colon cancer, a study using emulsion nanoformulations of curcumin and piperine observed that this combination additively contributed to the anticancer activity of curcumin in HCT116 cells [115]. 

In obese rats that were fed high fat diets (HFDs) (60% kcal), the concomitant administration of curcumin and salsalate (C/Sal) was better than either substance administered alone at suppressing pro-cancerous molecular pathways and colorectal tumorigenesis. In comparison to the HFD control, C/Sal suppressed activation of the phosphoinositide 3-kinase (PI3K), protein kinase B (Akt), rapamycin (mTOR) nuclear factor kappa B (NF-κB), and Wnt pathways, activated 5’ adenosine monophosphate (AMP)-activated protein kinase (AMPK), attenuated abnormal proliferation of the colonic mucosa, and reduced tumor multiplicity and burden [116]. Similarly, Wu et al. explored the effects of curcumin with or without Sal on inflammatory cytokines and pro-carcinogenic signaling in azoxymethane-treated A/J mice and found that the concomitant administration of C/Sal reduced the concentration of pro-inflammatory cytokines and decreased the activation of Akt and NF-κB more effectively than curcumin alone [117]. 

The combination of curcumin and vitamin B6 (C/B6) has also been investigated in the suppression of tumorigenesis induced by obesity. A study conducted by Wu et al. observed that C/B6 decreased fecal calprotectin and reduced tumor multiplicity and the total tumor burden, and also downregulated colonic phosphatidylinositol-4,5-bisphosphate 3-kinases (PI3Ks), Wnt, and NF-κB signaling [118].

Taken together, the findings show that nanotechnology-based systems and bioactive dietary compounds, like piperine and others for increasing the oral bioavailability of poorly bioavailable molecules such as curcumin, is an effective and promising strategy that can increase the bioactivity in cells.

#### 2.4.2. Regulatory Effects of Curcumin in the Liver and Adipose Tissue

As previously pointed out, the absorption of curcumin after oral intake is poor, and it is mostly excreted in the feces. The small, absorbed portion of the dose is metabolized and eliminated by biliary and renal excretion, and no accumulation occurs in organs [119,120]. It has also been shown that curcumin has metabolic instability. Most of the ingested oral curcumin is converted into water-soluble metabolites, glucuronides, and sulfates, thereby leading to low levels of free curcumin in the blood [119,121]. Thus, the beneficial effects of curcumin are in part mediated by its metabolites and/or degradation products [122]. Numerous efforts have been made to increase the oral bioavailability of curcumin, namely by developing formulations with adjuvants, nanoparticles liposomes, and micelles.

Regarding adjuvants, piperine, when administered concomitantly with 2 g of curcumin in healthy human volunteers, increased the curcumin bioavailability by 2000% [17]. Quercetin, administered with curcumin to five patients with familial adenomatous polyposis, decreased the number and size of polyps after a 6-month treatment with both substances [123]. 

Nanotechnology-based delivery systems such as micelles, liposomes, and polymeric, metal, and solid lipid nanoparticles have also been applied to enhance curcumin bioavailability [124].

Despite the issues related to its bioavailability, curcumin has innumerous benefits. It has shown potential as a therapeutic agent for liver diseases due to its anti-inflammatory, antioxidant, and antifibrotic properties. Lukita-Atmadja et al. showed that endotoxemic BALB/C mice previously treated with curcuminoids had reduced the phagocytic activity of Kupffer cells and suppressed hepatic microvascular inflammatory response to LPS. The authors hypothesized that curcumin’s anti-inflammatory effects may be mediated by the inhibition of the nuclear translocation of NF-κB and its dependent proinflammatory cytokines, which in turn contribute to decreased neutrophil recruitment to portal and central venules, as well as in the sinusoids [125].

Nanoformulated curcumin has been tested in liver diseases and the benefits include the increased efficacy of curcumin as a drug by maximizing its solubility and bioavailability, enhancing its membrane permeability, and improving its pharmacokinetics, pharmacodynamics, and biodistribution. Nanoformulation can overcome curcumin problems, such as low solubility and bioavailability, which can limit its therapeutic efficacy [120].

Jazayeri-Tehrani et al. examined the effects of nanocurcumin on overweight/obese non-alcoholic fatty liver disease (NAFLD) patients by assessing glucose, lipids, inflammation, insulin resistance, and liver function indices, especially through nesfatin. It was a double-blind, randomized, placebo-controlled clinical trial that included 84 overweight/obese patients with NAFLD diagnosed using ultrasonography. The patients were randomly divided into nanocurcumin and placebo groups. Interventions comprised two 40 mg capsules/day after meals for 3 months. The nanocurcumin group, when compared with the placebo, showed significantly increased high-density lipoprotein (HDL) cholesterol, insulin resistance, and decreased fatty liver degree, liver transaminases, waist circumference, hemoglobin A1C (HbA1c), triglycerides, total cholesterol, low-density lipoprotein (LDL), homeostasis model assessment of insulin resistance (HOMA-IR), TNF-α, high-sensitivity C-reactive protein (hs-CRP), and IL-6 [126]. 

Besides curcumin effects in the liver, adipose tissue has been studied as a possible target for curcumin as well. The growth and expansion of the adipose tissue requires angiogenesis. This is mediated by the secretion of adipokines, including leptin, adiponectin, resistin, visfatin, tumor necrosis factor-α, interleukin-6, interleukin-1, and vascular endothelial growth factor (VEGF). Ejaz et al. investigated the effect of curcumin added to the cell culture (adipocytes isolated from gonadal adipose tissue of mice) at concentrations of 0, 5, 10, or 20 µmol/L and incubated for 24 h. They showed that curcumin inhibited the formation of 3T3-L1 adipocytes. Further, they studied eighteen 4-week-old male C57BL/6 mice that were randomly assigned to three groups. The control group was fed the purified diet (AIN-93) containing 4% fat by weight; the high fat-fed group (HF) was fed an AIN-93 diet containing 22% fat by weight, and another group was fed the same HF diet supplemented with 500 mg of curcumin. The curcumin-supplemented group showed reduced liver weight and reduced hepatic steatosis [127].

Curcumin influenced signaling and gene expression of several lipid transport and metabolic genes leading to hypolipidemic effects [58]. LDL receptor knockout mice treated with increasing doses of curcumin (500 mg/kg of diet, 1000 mg/kg of diet, and 1500 mg/kg of diet for 16 weeks) showed marked decrease in body weight, liver weight, body fat, adipose tissue fat, and inflammatory markers, altogether explaining the reduction in high fat-induced atherosclerosis and steatohepatitis [64]. Besides, HFD and curcumin influenced cyclic adenosine monophosphate (cAMP) levels and CREB phosphorylation (ratio of pCREB)/CREB (cAMP response element-binding protein) and affected cluster of differentiation 36 (CD36) scavenger receptor/fatty acids translocase (CD36/FAT) and fatty acid binding protein 4 (FABP4/aP2) and uncoupling protein-1/2 (UCP-1/2) possibly as result of influencing CREB, nuclear factor erythroid 2–related factor 2 (NRF2), and peroxisome proliferator-activated receptor gamma (PPARγ) activity [63].

Interestingly, Zhao et al. demonstrated that 10 µM of curcumin added to 3T3-L1 preadipocytes accelerated basic mitochondrial respiration, adenosine triphosphate (ATP) production, and uncoupling capacity via the regulation of PPARγ. Also, curcumin increased the mRNA and protein expression of mitochondrial uncoupling protein 1 (UCP1), PPARγ, and peroxisome proliferator-activated receptor-γ coactivator-1α (PGC-1α) in vivo and in vitro [128].

Curcumin and piperine supplementation for obese mice under caloric restriction may increase the loss of body fat and suppresses HFD-induced inflammation. The study protocol consisted of three phases. During the 23-week period of phase 1, all 47 eight-week-old male C57BL/6 mice were fed a western style HFD (44% calorie from fat) to induce obesity. Mice were then divided into five groups. Group 1 continued consuming HFD ad libitum for the rest of study (30 weeks). The remaining four groups were submitted to caloric restriction, first by 10% for 10 weeks (phase 2), then by 20% (phase 3) for 20 weeks through the rest of the study. During phase 2 and 3, group 3 had HFD supplemented with a 1 g of curcumin/kg diet, group 4 supplemented with a 50 mg of piperine/kg diet, and group 5 with a 1 g of curcumin/kg diet + a 50 mg of piperine/kg diet. As a result, a significant reduction was observed in percent adiposity in the group under calorie restriction, supplemented with curcumin + piperine over a period of 20 weeks [129].

Bertoncini-Silva et al. studied C57BL/6 male mice fed a HFD with different doses of curcumin (50, 250, and 500 mg/kg body weight), and they observed that, in the liver, there was a reduction in TNF-α at all three doses and an increase in IL-10 at doses of 50 and 250 mg/kg body weight [130].

Di Pierro et al. studied the effect of curcumin in the form of phytosome in patients with obesity and metabolic syndrome, on body weight and waist circumference. This was a 2-month randomized, controlled clinical trial. Forty-four participants were randomly assigned to receive daily treatment with a curcumin-based product or pure phosphatidylserine for 30 days. The curcumin supplement was formulated to be enteric-coated and contained 800 mg/day of *Curcuma longa* extract, complexed with sunflower phospholipids (20% phosphatidylserine) and blended with 8 mg/day of piperine. Curcumin administration increased weight loss from 1.88 to 4.91%, enhanced percentage reduction in body fat (from 0.70 to 8.43%), and increased waist circumference reduction (from 2.36 to 4.14%) [131].

Clinical evidence remains inconclusive because of discrepancies regarding the optimal dosage, duration, and formulation of curcumin. A systematic review and meta-analysis of randomized controlled trials including 2038 participants support further investigation to confirm these data and to optimize the anti-inflammatory effects of curcumin oral supplementation in humans with chronic inflammation [112].

#### 2.4.3. Regulatory Effects of Curcumin in Heart and Vessels 

Curcumin has been shown to have potential as a therapeutic agent in various cardiovascular diseases due to its antioxidant, anti-inflammatory, anti-apoptotic, and lipid-lowering properties [132]. It has been reported to exert anticoagulant, anti-hypercholesterolemic, anti-hypertensive, and anti-atherosclerotic effects [133,134,135]. Additionally, curcumin has been proven to reduce the consequences of myocardial infarction, cardiac ischemia, and reperfusion injury [136]. The many molecular targets involved in its actions include NF-κB, angiotensin II type receptor (AT1R), nuclear factor erythroid 2 (NFE2)-related factor 2 (NRF2), toll-like receptor 4 (TLR4), and SIRT3-mediated signaling pathways [137].

However, in clinical applications, curcumin faces significant challenges, primarily due to its poor bioavailability (Table 1). In recent years, the development of curcumin-loaded nanoformulations has helped overcome some of its pharmacokinetic limitations, leading to more favorable outcomes in the field of cardiovascular disease. Preclinical and clinical studies have tested the efficacy of these nanodrug delivery systems.

For instance, curcumin encapsulated in carboxymethyl chitosan nanoparticles conjugated to a myocyte-specific homing peptide showed higher cardiac bioavailability of the phytocompound at a low dose of 5 mg/kg body weight, compared to free curcumin at 35 mg/kg body weight [138]. Additionally, the nanoformulation efficiently improved cardiac function by downregulating the expression of hypertrophy marker genes (atrial natriuretic factor [ANF], β-myosin heavy chain [β-MHC]), apoptotic mediators (Bax and cytochrome-c), and activity of apoptotic markers (caspase 3 and polymerase), whereas free curcumin at a much higher dose provided minor benefits. By enhancing the aqueous-phase solubility and tissue bioavailability of curcumin, diverse nanocurcumin formulations such as hyaluronic acid-based nanocapsules, nanoparticles encapsulated in poly(lactic-co-glycolic acid) (PLGA) or nanoemulsion systems were also proven to have a net benefit in preventing and treating hypertension and its complications in rodents and in in vitro studies [139,140,141,142].

Free curcumin has been reported to have beneficial effects against several cardiac arrhythmias [143,144,145,146], attributed, among other actions, to its antioxidant properties [147]. It has also been demonstrated that an encapsulated lipopolymeric hybrid nanoparticle formulation of curcumin, while enhancing the bioavailability and stability of the compound, could protect against QT prolongation by activating the hERG (human ether-a-go-go-related gene) K+ channel, in a manner superior to liposomal curcumin [148]. 

In several animal studies on isoproterenol-induced myocardial infarction (MI), curcumin exerted its beneficial actions by stabilizing the lysosomal membranes, reducing the release and activity of lysosomal enzymes, and scavenging ROSs generated during the ischemic process [149,150]. Interestingly, Boarescu et al. reported that nanocurcumin polymer-based nanoparticles better prevented rat myocardial damage extension, reduced interstitial edema and electrocardiogram alterations, and were more powerful for enhancing myocardial antioxidative capacity than conventional curcumin [151]. In another study, formulated curcumin and nisin-based poly lactic acid nanoparticles administered before an isoproterenol-induced MI lesion in guinea pigs resulted in the prevention of post-MI atrial fibrillation and decreased levels of cardiac troponin I and kidney injury molecule-1 [152]. The formulated nanoparticle conferred a significant level of cardio-protection and was nontoxic.

Curcumin was effective against ischemia/reperfusion (I/R) lesions, as well in various experimental models, primarily through antioxidant actions such as scavenging ROSs [153], increasing mitochondrial superoxide dismutase (SOD) activity and decreasing the generation of MDA [154], or by regulating mitochondrial dysfunction [154]. Anti-inflammatory [155,156], antiapoptotic [157], and autophagy regulatory effects [158] were also reported. Once again, curcumin nanoformulations were suggested to possess therapeutic usefulness [159]. 

The few clinical trials conducted to investigate the role of curcumin for mitigating the I/R lesion effects have yielded contradictory outcomes. In their studies, Aslanabadi et al. [160] and Phrommintikul et al. [161] administered a short course of curcumin treatment (1–2 days), orally, as nano-micelle or in capsule form respectively, with a low total dosage of 0.48 g–8 g. They found no statistical significance between the curcumin group and the control group in terms of blood myocardial injury biomarkers (hs-cTnT, cTnI, creatine kinase muscle and brain type (CK-MB)) after percutaneous coronary intervention (PCI). Interestingly, a longer course of treatment (8–10 days) with a higher dosage (32 g–40 g in total) revealed that conventional curcuminoids administered orally in capsules might exert cardioprotective effects in terms of anti-inflammation, antioxidant activity, cardiac function, and incidence of in-hospital myocardial infarction [162]. However, in 2021, a clinical trial conducted on patients undergoing elective coronary angioplasty reported the superior effects of nanocurcumin (80 mg/capsule) versus free curcumin (500 mg/capsule), administered daily for 8 weeks, in reducing total cholesterol (TC), triglycerides (TG), TNF-α, and MDA and increasing SOD levels [163]. 

Improving risk factors for cardiovascular disease (CVD), such as obesity, hypertension, diabetes, metabolic syndrome, and dyslipidemia, is a widely addressed target, mostly by a multitude of pharmacotherapies. Given the numerous side effects of medications, nutraceutical treatments are gaining interest as alternative or adjunct treatments, with curcumin as a promising candidate. A recent meta-analysis included nine randomized controlled clinical trials that investigated the effects of nanocurcumin supplementation on risk factors for CVD, conducted up to 2021 [43]. Fasting glucose, insulin, and HOMA-IR were significantly decreased in the nanocurcumin group, and high-density lipoprotein (HDL) increased, whereas TG, TC, and LDL levels were improved more substantially in subjects with dyslipidemia at baseline. In addition, C-reactive protein (CRP) and interleukin-6 (IL-6) were decreased following nanocurcumin supplementation, showing efficient anti-inflammatory effects. A decrease in systolic blood pressure was also reported in the treatment group. In line with this, a clinical trial conducted in 2023 by Dastani et al. reported protective anti-inflammatory and anti-atherosclerotic effects of nanocurcumin (administered for 90 days, 80 mg/day) in type 2 diabetes mellitus patients with mild to moderate coronary artery disease (CAD) [164]. Another trial performed in hemodialysis patients, known as a high-risk population for cardiovascular disease, provided evidence that nanocurcumin (120 mg/day, 12 weeks) decreases the serum levels of CRP, along with vascular cell adhesion molecule 1 (VCAM-1) and intercellular adhesion molecule 1 (ICAM-1), two proinflammatory adhesion molecules involved in endothelial dysfunction, but had no significant effect on plasma lipid profile [165]. It is worth noting that conventional curcumin administered to coronary artery patients had modest outcomes. In a pilot, randomized, double-blind, placebo-controlled trial, the curcumin group experienced a decrease in serum levels of triglycerides, LDL-cholesterol, and VLDL-cholesterol compared to the baseline values [166]. However, there were no significant improvements observed in total cholesterol, HDL, blood glucose, or hs-CRP levels.

Overall, the effectiveness of curcumin nanoformulations in preventing and treating various cardiovascular diseases, although very promising, requires further investigation, especially in clinical settings.

#### 2.4.4. Regulatory Effects of Curcumin in Brain and Cognitive Disorders

Curcumin has poor bioavailability, especially in the brain, where the BBB further limits its absorption. However, significant efforts have been made over the past decade to develop pharmacological strategies that significantly increase its uptake in the brain, with promising results in pathological models. For example, formulations of curcumin complexed with galactomannans, which have better BBB penetration than unformulated curcumin [167], have shown beneficial effects against neuroinflammation, anxiety, fatigue, and memory loss, in both humans and animal studies [168,169,170,171]. Loading curcumin into natural nanoparticles such as exosomes or liposomes has also been proven effective. In experimental models of brain injury, intranasal administration of curcumin-laden exosomes reduced neuroinflammation and cancer progression [172], while their intravenous injection exerted neuroprotective effects during cerebral ischemia by abolishing the generation of ROSs, reducing apoptosis and attenuating BBB disruption [173]. Formulations with liposomes have been described as less effective than those with exosomes [103]; however, in animal and cellular models of Alzheimer’s disease (AD), treatment with curcumin-laden liposomes has shown some anti-amyloidogenic and anti-inflammatory efficacy [174]. 

Neuroinflammation and memory loss, along with the accumulation of misfolded proteins and oxidative stress, are common features of neurodegenerative disorders such as AD, the incidence of which is increasing worldwide due to increasing life expectancy. Given the large number of studies suggesting that curcumin treatment can reduce all major pathological markers of AD, we believe this topic is worth exploring further.

The widely held dogma identifies amyloid-β (Aβ) as the primary culprit in AD. According to the so-called amyloid cascade hypothesis [175], cerebral Aβ accumulation leads to a series of events consisting of plaque formation, the aggregation of hyperphosphorylated tau protein in paired helical filaments (PHFs) and neurofibrillary tangles (NFTs), neuronal death, and concomitant inflammatory response. 

Regarding the production of Aβ, studies of in vitro and mouse models have shown that curcumin inhibits BACE1, an enzyme involved in the amyloidogenic cleavage of the Aβ precursor protein (APP) [176,177]. Furthermore, curcumin appears to reduce the production of Aβ also by affecting a second enzyme required for the cleavage of APP, the glycogen synthase kinase-3 beta (GSK3β)-dependent presenilin 1 (PS1) [178]. Interestingly, in addition to activating PS1 and among various other functions, GSK3β kinase phosphorylates tau protein at several residues. Given that the over-activation of GSK3β has been linked to tau aggregation into paired helical filaments (PHFs) and neurofibrillary tangles (NFTs) [179], the inhibition of GSK3β by curcumin would hinder both Aβ production and tau aggregation, two crucial events in AD pathogenesis.

In 2012, Dong et al. showed that the prolonged treatment of aged rats with curcumin stimulates neurogenesis in the hippocampus, a brain region affected early in AD and deeply involved in the process of new memory formation. Consistently, the authors also found that most of the genes regulated by curcumin have biological and physiological implications in brain development and cognitive function [180]. 

In line with these observations, chronic curcumin administration improved memory acquisition and consolidation in both adult and aged rats [181], while curcumin–galactomannan complex supplementation offered a significant reduction in spatial memory impairment [167].

Unfortunately, despite the promising results obtained in preclinical models, only a limited number of studies have investigated the effects of curcumin in AD clinical trials. The first of these studies was published in 2008 and found no changes between the curcumin and placebo groups in terms of plasma Aβ and tau levels [182]. Moreover, the lack of cognitive decline in the placebo group in this 6-month trial precluded the detection of a protective effect of curcumin [182]. A subsequent 24-week randomized, double-blind, placebo-controlled study on thirty-six people with mild to moderate AD showed no differences between treatment groups in biomarker or clinical efficacy measures [183]. On the other hand, results from a more recent 18-month placebo-controlled trial involving non-demented adults revealed that daily oral administration of curcumin improves memory and reduces the accumulation of amyloid and tau in the amygdala and hypothalamus [184]. 

The discrepancies between the different studies may be due to various factors, such as the choice of the experimental model (animal vs. human), the formulation of curcumin (more or less bioavailable), its dosage, and the duration of treatment.

### 2.5. Potential Safe Concentration Range and Toxicity of Curcumin 

Curcumin has been demonstrated to be non-toxic [185]. Studies performed in vitro, on animals, and in humans regarding the toxicity of curcumin have confirmed its safety [186]. In animal studies, no toxicity has been observed from a dose of approximately 1.2 g curcumin/kg body weight administered for 14 days [20] and also with doses of 300 mg of curcumin/kg body weight for 14 weeks [26]. Human trials also describe a safe concentration range with no toxicity using doses varying from 1125 to 2500 mg of curcumin/day [186]. A study of high doses of oral curcumin up to 8 g daily for three months observed no toxicity in patients with pre-invasive malignant or high-risk pre-malignant conditions [21].

The efficacy and toxicity of curcumin have also been investigated through its use as nanoparticles (curcumin-NPs) for treatment, with the purpose of enhancing tumor growth suppression and reducing adverse effects. A systematic review of in vivo models of breast cancer showed that all the studies that evaluated the toxicity of curcumin-NPs found them to be safe regarding hematological and biochemical markers, as well as damage to major organs [187].

Besides being safe and non-toxic, curcumin has been investigated to promote cardioprotective effects against chemotherapy-induced cardiotoxicity [188]. In a rat embryonic cardiomyocytes model of doxorubicin-induced cardiotoxicity, curcumin co-loaded with resveratrol (5:1) in Pluronic F127 micelles led to a 1617-fold increase in the aqueous solubility of curcumin, compared to the drug alone [189]. The curcumin/resveratrol formulation proved efficient in exerting protective antioxidant and antiapoptotic effects in H9C2 cells exposed to the chemotherapeutic treatment. In vivo, a recent meta-analysis reported higher protective effects of curcumin nanoformulations against doxorubicin-induced cardiotoxicity in rodents when compared to free curcumin, mainly through antioxidant, anti-inflammatory, and antiapoptotic actions [190]. Likewise, reduced toxicity following curcumin treatment was reported in hydroxyapatite-injured rat hearts. The compound, administered as nanoparticles with a crystalline structure and an average size of 0.04 μm, acted mainly by restoring the activity of antioxidant enzymes [191]. Similar protective effects of curcumin and its nanoformulations have been reported in several tissues in patients receiving chemotherapy and radiotherapy [192].

## 3. Conclusions

Progress in nanoscience has provided tools to overcome the insolubility issues associated with hydrophobic molecules, such as curcumin. Nanoformulations based on curcumin examined in this review efficiently address a range of signaling pathways associated with diverse human diseases, often leveraging antioxidant properties. They not only enhance the dispersion of curcumin in aqueous solutions, but also present significant advantages compared to conventional delivery methods. These advantages encompass versatile surface properties, adjustable particle size, and the capability to modify the pharmacokinetics and therapeutic attributes of curcumin, ultimately achieving a more potent therapeutic effect. Additionally, the utilization of curcumin-based nanomedicines with the aforementioned abilities holds promise in medical theranostics, particularly in addressing the individualized pharmacological needs of patients with inflammatory, immune-related, or degenerative diseases.

The majority of the published evidence on the topic addressed here is pre-clinical, consistently showing the enhanced efficacy of curcumin-based nanoformulations in improving curcumin’s bioavailability and bioactivity. Comparative interventions between conventional curcumin and nanocurcumin formulations supported this conclusion across cell, animal, and also human studies. Future investigations on large human cohorts are required to establish efficient dosages and time courses of administration for various curcumin nano-therapies. Combination therapy of curcumin in nanoparticles is another beneficial approach that proves very promising for enhancing curcumin bioactivity and stability, thereby reducing the daily dose of the phytocompound and the associated treatment costs. Additionally, there is a need for in-depth exploration through comprehensive studies into the bio-interactions between cellular structures and curcumin nanoparticles, to gain a better understanding of the mechanisms involved in their selective uptake.

## Figures and Tables

**Table 1:** Levels of curcumin in various organs and tissues.

Method of Administration	Dose	Time	Levels of Curcumin	Local	Model	Reference
Oral	0.1 g/kg	1 h later	0.22 µg/mL	Plasma	Mice	[12]
Oral	0.1 g/kg	6 h later	5 ng/mL	Plasma	Mice	
Oral	340 mg/kg	2 h later	16.1 ng/mL	Plasma	Rat	[16]
Oral	340 mg/kg	2 h later	1.4 mg/g	Intestinal mucosa	Rat	[16]
Oral	1.2 g/kg	-	0–12 nM	Plasma	Rat	[20]
Oral	1.2 g/kg	-	0.2–1.8 µmol/g	Colon mucosa	Rat	[20]
Oral	2 g/kg	Within 0.83 h	1.35 µg/mL	Serum	Rat	[17]
Oral	2 g/kg	1 h later	1.00 µg/mL	Serum	Rat	[9,17]
Oral	2 g/kg	1 h later	0.006 µg/mL	Serum	Human	[17]
Oral	2.35 g/day	40 h later	127.8 nmol/g	Intestinal mucosa	Human	[28]
Oral	3.6 g/day	1 h later	11.1 nmol/L	Plasma	Human	[22]
Oral	3600 mg/day	38 min later	12.7 nmol/g	Colorectum	Human	[27]
Oral	4 g/day	Within 12 h	0.51 µM	Serum	Human	[21]
Oral	6 g/day	Within 12 h	0.63 µM	Serum	Human	[21]
Oral	8 g/day	Within 12 h	1.77 µM	Serum	Human	[21]
Oral	Up to 8 g/day	After 1, 2, or 4 h	Remained undetectable	Serum	Human	[18,19]
Oral	10 g/day	After 1, 2, or 4 h	50.5 ng/mL	Serum	Human	[18,19]
Oral	12 g/day	After 1, 2, or 4 h	51.2 ng/mL	Serum	Human	[18,19]
Intraperitoneal	0.1 g/kg	15 min later	2.25 µg/mL	Plasma	Mice	[12]
Intraperitoneal	0.1 g/kg	1 h later	177.04 µg/g	Intestine	Mice	[12]
Intraperitoneal	100 mg/kg	2 h later	200 nmol/g	Intestinal mucosa	Mice	[26]
-	5 µM	15 min later	0.013 µM	Caco-2 cells	In vitro	[29]
-	5 µM	60 min later	0.055 µM	Caco-2 cells	In vitro	
-	5 µM	4 h later	0.031 µM	Caco-2 cells	In vitro	
-	10 µM	10 min later	1313 pmol/2.0 × 10^6^	THP-1 monocytes and macrophages	In vitro	[30]
		2 h later	2029 pmol/2.0 × 10^6^			
		24 h later	401 pmol/2.0 × 10^6^			
-	25 µM	1–9 h later	1840–5650 pmol/2.5 × 10^6^	HepG2 cells	In vitro	[11]

**Table 2:** Major curcumin nanoformulations and their applications.

Applications/Target Tissues	Curcumin Nanoformulations (Examples)
Antimicrobial activities (viruses, bacteria, fungi)	Colloidal (micelles, liposomes, nanoemulsions, cyclodextrins, chitosan, and polymeric nanoparticles) metallic and mesoporous particles, graphene, quantum dots, and hybrid nanosystems such as films and hydrogels [35].
Wound healing	Nanofibers, nanoparticles, nanomicelles, nanofibers, films, composites, scaffolds, gels and hydrogels, sponges, and aerogels [36].
Inflammatory and oxidative stress-related diseases	Liposomes, polymeric micelles, Metal organic frameworks (MOFs), inorganic nanocarriers, polymeric nanoparticles, proteins, and nanofibers [34,37,38].
Gastrointestinal	Solid dispersions, nano/microparticles, polymeric micelles, nanosuspensions, lipid-based nanocarriers, cyclodextrins, conjugates, and polymorphs [37].
Liver	Phosphatidylserine (PS)-modified nanostructured lipid carriers (mNLCs), Gold nanoparticles (AuNPs), Cur hyaluronic acid–polylactide nanoparticles (HPNPs), Silver nanoparticles (AgNPs), and simple nano curcumin (nanoCUR) [39,40].
Adipose tissue	Curcuminoid nanomicelles for adipose-derived mesenchymal stem cells [41].
Cardiovascular	Curumin-Bioperin PLGA NPs (type of nanoformulation), nano curcumin, nanoparticles, and lipososmes [39,42,43].
Lung	Curcumin-loaded phospholipid vesicles, polymer glycerosomes, and liposomal formulations [38,40].
Brain	Curcumin-loaded PLGA nanoparticles (C-NPs) [44].
Cancer (e.g., breast)	Curcumin and resveratrol in liposomes, polymeric micelles and phospholipid complexes, polymeric nanoparticles, dendrimers, adjuvants, solid lipid nanoparticles, nanosponges, nanoemulsions, and nanogels [45,46].
Mitochondria	Mitochondria-targeted polymeric nanoparticle (NP) system [47].
